# Predicting venous thromboembolism in hospitalized trauma patients: a combination of the Caprini score and data-driven machine learning model

**DOI:** 10.1186/s12873-021-00447-x

**Published:** 2021-05-10

**Authors:** Lingxiao He, Lei Luo, Xiaoling Hou, Dengbin Liao, Ran Liu, Chaowei Ouyang, Guanglin Wang

**Affiliations:** 1 Trauma Center of West China Hospital/West China School of Nursing, Sichuan University, Guo Xue Road 37#, Chengdu, 610041 China; 2 College of Chemical Engineering, Sichuan University, Chengdu, China; 3 Engineering Research Center of Medical Information Technology, Ministry of Education, West China Hospital of Sichuan University, Chengdu, China; 4 Trauma Center of West China Hospital/West China School of Medicine, Sichuan University, Guo Xue Road 37#, Chengdu, 610041 China

**Keywords:** Venous thromboembolism, Wounds and injuries, Risk assessment, Machine learning, Models, statistical

## Abstract

**Background:**

Venous thromboembolism (VTE) is a common complication of hospitalized trauma patients and has an adverse impact on patient outcomes. However, there is still a lack of appropriate tools for effectively predicting VTE for trauma patients. We try to verify the accuracy of the Caprini score for predicting VTE in trauma patients, and further improve the prediction through machine learning algorithms.

**Methods:**

We retrospectively reviewed emergency trauma patients who were admitted to a trauma center in a tertiary hospital from September 2019 to March 2020. The data in the patient’s electronic health record (EHR) and the Caprini score were extracted, combined with multiple feature screening methods and the random forest (RF) algorithm to constructs the VTE prediction model, and compares the prediction performance of (1) using only Caprini score; (2) using EHR data to build a machine learning model; (3) using EHR data and Caprini score to build a machine learning model. True Positive Rate (TPR), False Positive Rate (FPR), Area Under Curve (AUC), accuracy, and precision were reported.

**Results:**

The Caprini score shows a good VTE prediction effect on the trauma hospitalized population when the cut-off point is 11 (TPR = 0.667, FPR = 0.227, AUC = 0.773), The best prediction model is LASSO+RF model combined with Caprini Score and other five features extracted from EHR data (TPR = 0.757, FPR = 0.290, AUC = 0.799).

**Conclusion:**

The Caprini score has good VTE prediction performance in trauma patients, and the use of machine learning methods can further improve the prediction performance.

**Supplementary Information:**

The online version contains supplementary material available at 10.1186/s12873-021-00447-x.

## Introduction

Venous thromboembolism (VTE), including deep vein thrombosis (DVT) and pulmonary embolism (PE), is a critical cause of death and disability in hospitalized patients. It is believed that around 5% ~ 10% of hospitalized deaths are caused by PE [1]. In the United States, there are more than 600,000 cases of symptomatic VTE per year, of which 2/3 are hospital-acquired and VTE-related deaths are about 296,000 cases per year [2]. Complications secondary to VTE, such as pulmonary hypertension, post-thrombotic syndrome, VTE recurrence, can also cause a huge health burden and long-term impact on the quality of life of patients [3, 4].

A combination of multiple factors leads to VTE, including gender, age, hospitalization, surgery, trauma, fracture, cancer, paralysis, cardiovascular or cerebrovascular events, etc. [5]. Specifically, trauma events usually contain the three main causes of thrombosis proposed by Virchow: blood flow stasis, blood hypercoagulability, and vascular endothelial injury, leading to the fact that the impact of trauma on the body is second only to surgery and is an independent risk factor for the occurrence of VTE [6].

Predicting the risk of VTE can guide prevention. Risk prediction tools for VTE prediction in trauma populations are often based on literature review and clinical experience, such as the Risk Assessment Profile (RAP) [7, 8] proposed by Greenfield L.J. in 1997, and the latter two simplified RAP scoring tools [9, 10] based on it, by which the scoring items selected are not inconsistent. In 2012, Rogers F.B. et al. retrospectively analyzed trauma-related VTE risk factors in 16,608 trauma patients, then formed the Traumatic Thrombosis Score System (TESS) [11]. However, in 2015, a study conducted by Zander A.L. et al. [12] shows that the prediction using both TESS and RAP does not get ideal results (TESS: sensitivity = 0.49, specificity = 0.72, AUC = 0.66; RAP: sensitivity = 0.83, specificity = 0.37, AUC = 0.66), revealing that the validity of VTE prediction tools are still inconsistent and not repeatable.

As a general VTE risk assessment scale in the surgical population, the Caprini risk assessment model (Caprini RAM) proposed by Caprini J.A. et al. in 1991 [13] has verified its application effects in more than 200 publications. It has been successfully received and recommended for surgical patients by the American College of Chest Physicians Association (ACCP) [14] and guidelines in some regions of China [15, 16]. The Caprini RAM has already taken many risk factors of hospitalized patients into account including fractures and some other trauma-related factors, but except in hip and lower limb fractures [17–20], there is still no definitive evidence proving its effectiveness for trauma populations. This paper will try to verify the accuracy of the Caprini RAM in predicting trauma-related VTE.

The empirical scale models usually based on the unary linear relationships between the variables and set score weights according to the relative thrombosis risk reported in the literature. However, there are several problems. On the one hand, there may be inaccuracies in estimating the effect based on experience; on the other hand, the weights of influencing factors may be inconsistent in different specific disease groups. In addition, linear scoring tools could not assess the interaction between multiple influencing factors accurately. In contrast, data-driven modeling methods can optimize the combination of all feature weights to the greatest extent, to better tap the potential of data prediction.

Machine learning methods help to improve the effect of linear predictions made by some traditional risk assessment models. For example, Wang X. et al. [17] compare the prediction effects of different machine learning models with the PADUA risk assessment scale in medical patients, finding out that machine learning model, especially the random forest model (RF), can obtain better prediction performance. Besides, researchers have made successful predictions for asymptomatic VTE events based on natural language processing and machine learning methods [18]. The results show that the key features for accurately predicting VTE can be extracted from text description factors, and machine learning may provide a new way for prediction and identification. However, in these papers, the data especially the medical history assessment and laboratory examination results have not been fully utilized, which should reflect multiple aspects of the medical system in detail and are more important for the modeling.

Medical data usually come from diverse sources and often possess attributes such as high dimension, nonlinearity, and imbalance in positive and negative samples. In machine learning, traditional methods such as multivariate linear regression and logistic regression (LR) may come to problems like heavy computational burden and multicollinearity in feature selection, which can be overcome by adding regularization and loss function settings under the premise of a linear model. Meanwhile, the mutual information entropy (MIE) algorithm based on information theory can also realize the selection through the calculation of the joint probability density function. In practice, which method should be chosen from the above is often determined by whether the data characteristics meet the prerequisites of a certain one [21]. Since there is no relevant research so far confirming which method is the most helpful for improving the clinical prediction model, in this paper, we will try to use different feature selection methods combined with a machine learning model to extract features that relate to VTE. A large number of electronic healthcare records (EHR) will be used for prediction modeling, including medical history evaluation and laboratory examination results.

In summary, this paper will try to achieve effective prediction for VTE in the trauma hospitalized population, which includes three parts: (1) Empirical modeling: verify the prediction effect of the empirical Caprini RAM for the trauma population; (2) Data-driven modeling: extract effective feature groups from the EHR data with different methods, then build different data-driven prediction models and compare them with the former empirical Caprini RAM; (3) Combine the Caprini RAM from part (1) with the best features group selected from part (2) to form a new machine learning model to obtain the best performance for the VTE prediction.

## Methods

### Study population and data collection

The West China Hospital Research Ethics Committee approved the study, all methods were performed in accordance with relevant guidelines, and informed consent was waived due to the retrospective nature of the study. The information of emergency trauma patients hospitalized in the Trauma Center of West China Hospital (a Tertiary hospital in Sichuan Province) were reviewed from September 2019 to March 2020 and obtained relevant data in the patient’s EHR.

The inclusion criteria in this study contain: (1) Days from injury to admission < 30; (2) age ≥ 18-year-old; (3) length of stay > 3 days; The exclusion criteria is: diagnosed with VTE on admission. The outcome indicator is the VTE that occurred during the patients’ hospitalization, including DVT and PE. The patient should get routine color duplex ultrasonography after admission and surgery, or any time found symptoms of limb swelling during hospitalization; If the patient has symptoms such as dyspnea, chest pain, decreased pulse oximetry, or abnormal arterial blood gas, Computed Tomography Pulmonary Angiography (CTPA) is used to diagnose PE.

All patients have to get VTE risk assessment on the day of hospitalization, which follows the widely accepted Caprini RAM version 2005 [22], then clinicians use low molecular weight heparin (LMWH) for chemoprophylaxis by risk stratification and clinical judgment.

In this paper, all the features for the VTE prediction are extracted from the EHR data, including 10 basic indicators, 18 medical history and comorbidity indicators, 24 laboratory examination indicators, 5 vital signs indicators, and 12 trauma-related indicators, which form a dataset containing 69 potential predicting factors (details are shown in additional file 1). Among these indicators, vital signs indicators and laboratory test indicators are based on the earliest results obtained after admission.

### Statistical methods and machine learning algorithms

All the EHR data extracted from the hospital information system are saved in Comma-Separated Values (CSV) format. Software such as Python 3.7, PyCharm IDE as well as python packages like Pandas, NumPy, and scikit-learn are adopted for data processing and modeling. For categorical indicators, One-Hot encoding is applied; while for numerical indicators, original values are kept. Missing values are implemented by the mean values. Due to the high dimensionality of the EHR data, multiple methods including Least absolute shrinkage and selection operator (Lasso) regression, Ridge regression, ElasticNet regression, Logistic regression (LR), Mutual Information Entropy (MIE) as well as Recursive Feature Elimination (RFE) are combined to filter the indicators into different feature groups for building the VTE prediction model.

The principle of Lasso regression, Ridge regression, and ElasticNet regression is to reduce the magnitude of the corresponding feature coefficients to eliminate irrelevant features and avoid over-fitting of the model by introducing a regularization term in the fitting objective function. Among these methods, the Lasso regression takes L1 norm regularization [23] and Ridge regression takes L2 norm regularization [24], while ElasticNet regression is a combination of the former two regularization terms [25]. This combination can maintain the sparse results of Lasso regression and inherit the stability of Ridge regression. Another feature screening method MIE considers the influence of indicator *X* on the distribution of target *Y*, i.e. VTE in this paper, as the correlation strength between these two variables. The information entropy *H*(*X*) represents the amount of information contained in a random variable *X* itself. When there is a correlation between two variables *X* and *Y*, the addition of *X* will significantly reduce the uncertainty of *Y,* thus the correlation strength between variable *X* and *Y*, *I*(*X; Y*), can be calculated as *I*(*X; Y*) = *H*(*X*) - *H*(*X*|*Y*), which is also called as mutual information entropy between *X* and *Y* [26]. A larger MIE value means a stronger correlation between *X* and *Y*, and thus *X* should be more likely to be taken into the prediction model for *Y*.

Once the feature screening is completed, different feature groups obtained by the above screening methods are selected to build the prediction models for VTE. This paper adopts the random forest (RF) model based on decision tree for the prediction modeling because it has better performance on high dimensional and unbalanced data sets with outliers. It can also achieve higher accuracy and better generalization ability compared with other machine learning algorithms and is widely used in medical predictions [27–29]. Additionally, the K-fold method is also adopted for internal validation. The fold number *k* and repeat times *r* are set to 10, 10, which means, for each model, a 10-fold validation test is conducted 10 times to reduce the type I errors [30]. Finally, for each model, average values of prediction metrics are obtained from the test set results including True Positive Rate (TPR, Recall, Sensitivity), False Positive Rate (FPR, 1-Sepcifity), AUC, Accuracy and Precision, and then compared with the metrics of Caprini RAM.

## Results

### Patient characteristics

**Fig. 1:**
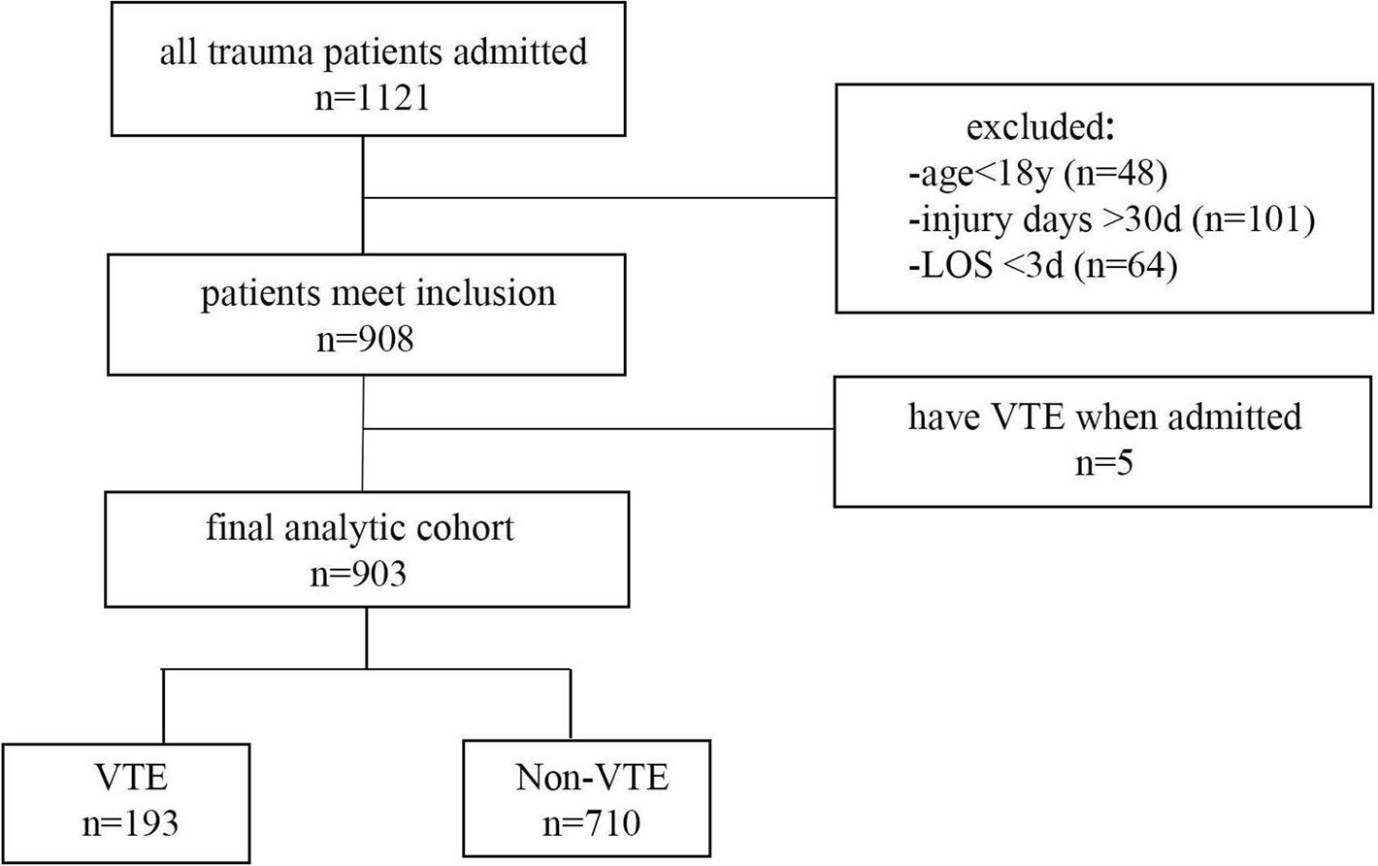
A Flowchart of the inclusion and exclusion process for the EHR data

The data of 1121 trauma patients admitted to the emergency department from September 2019 to March 2020 is extracted from the EHR. Finally, only 903 patients met the inclusion and exclusion criteria. The inclusion and exclusion process are listed in Fig. 1.

The demographics of the 903 patients are shown in Table 1, among which 561 are males while the other 342 are females. The medians of age, Injury Severity Scale (ISS), the Caprini scores, and BMI are 51, 9, 9, and 23.35 ± 3.62, respectively. 193 patients developed VTE, including 187 cases with DVT and 6 cases with both DVT and PE. The missing rates of all the fields in the EHR data are low, of which the highest is 5.2% and corresponds to the Body Mass Index (BMI) (additional file 1).

**Table 1:** Patients Demographics^a^

Demographics	Total(***N*** = 903)	VTE(***N*** = 193)	Non-VTE(***N*** = 710)
**Gender**			
Male	561 (62.13%)	115	446
Female	342 (37.87%)	78	264
**Age**	51 (38 ~ 65)	56 (46 ~ 73)	49 (36 ~ 62)
**ISS**	9 (5 ~ 20)	13 (9 ~ 26)	9 (4 ~ 16)
**BMI**	23.35 ± 3.62	24.05 ± 4.27	23.17 ± 3.41
**Mechanism of Injury**			
Blunt	871 (96.46%)	190	681
Penetrating	32 (3.54%)	3	29
**Injury Cause**			
Crush injury	55 (6.09%)	11	44
Fall injury	324 (35.88%)	62	262
High fall injury	165 (18.27%)	49	116
Firearm injury	5 (0.55%)	2	3
Machine injury	50 (5.54%)	5	45
Sharp injury	15 (1.66%)	1	14
Traffic accident	250 (27.69%)	60	190
Other	39 (4.32%)	3	36
**Surgery**			
Yes	879 (97.34%)	187	692
No	24 (2.66%)	6	18
**Central venous access**			
Yes	50 (5.54%)	24	26
No	853 (94.46%)	169	684
**Caprini Score**	9 (5 ~ 11)	11 (10 ~ 12)	8 (4 ~ 10)
**Chemoprophylaxis**			
LMWH Use	642 (71.10%)	173	469
**Length of Stay**	12 (8 ~ 19)	18 (11 ~ 30)	10 (7 ~ 16)
**Death in Hospital**	4 (0.44%)	1	3
**Days after Injury for VTE diagnosis**	NA	11 (5 ~ 17)	NA

^a^ Note: categorized data is described by “*n*(%)”; data conforming to the normal distribution is described by “*x* ± *s*”; data that does not conform to the normal distribution is described by the median and quartile; Abbreviations: *ISS* Injury Severity Score. *LMWH* Low Molecular Weight Heparin. *NA* not applicable

### Model metrics of the Caprini RAM

**Fig. 2:**
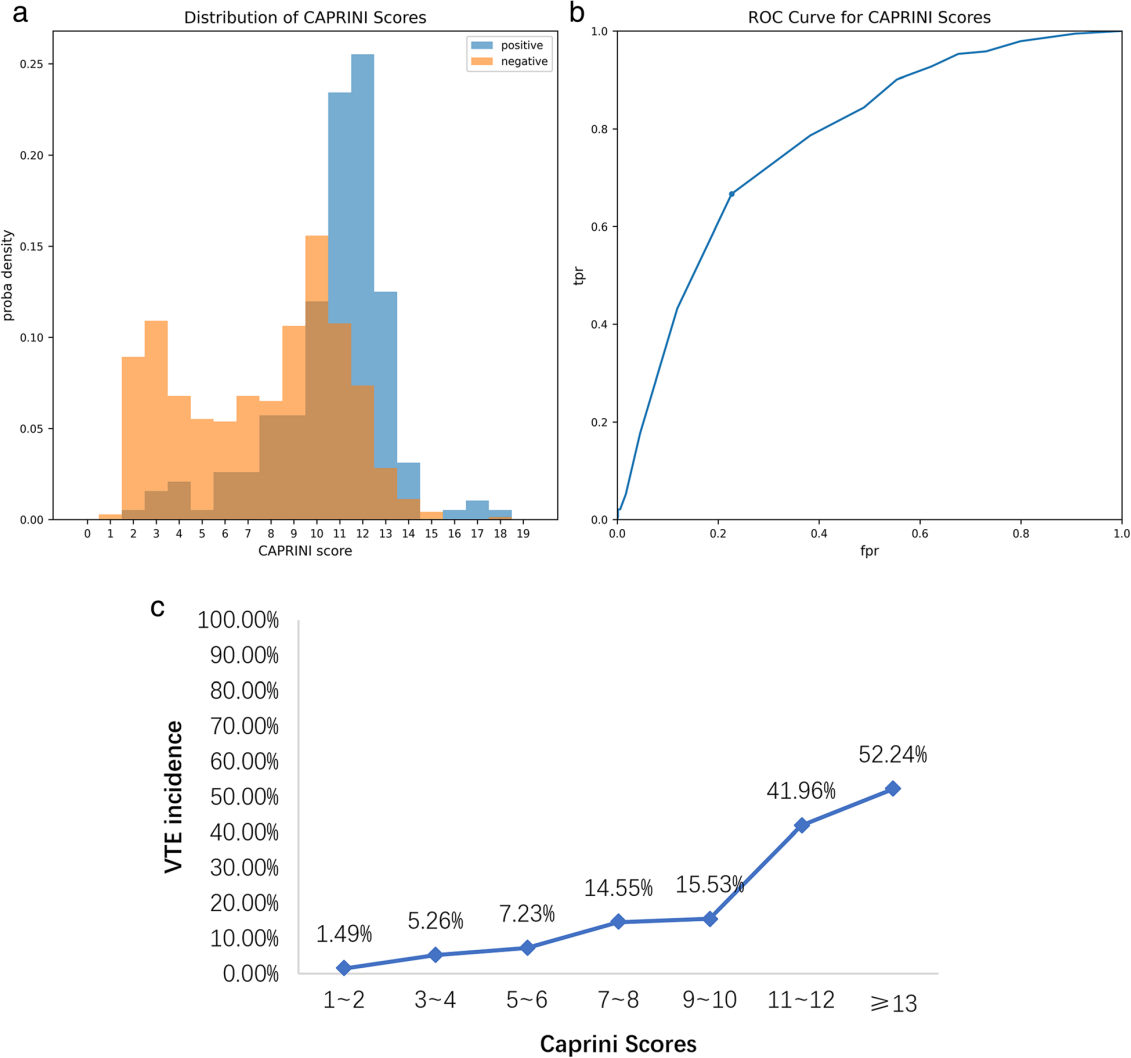
(**a**) Caprini scores distribution for the VTE and non-VTE patients; (**b**) receiver operating characteristic (ROC) curve of the Caprini RAM. (**c**) VTE incidence curve of the Caprini RAM

The total Caprini scores distributions for the 903 patients are plotted in Fig. 2a, scores range from 1 to 18 points, among which the VTE group scores distribution is 11 (10 ~ 12) points, while for the non-VTE patients the distribution is 9 (5 ~ 11) points. Comparing the incidence in different scoring segments shows that the Caprini score increases as the risk grows (Fig. 2c). This Caprini RAM gets AUC = 0.773 with the best cut-off score = 11 points (Fig. 2b), corresponding model metrics are listed in Table 2.

**Table 2:** Prediction Metrics of Different Models based on EHR Data (Compared with the Caprini RAM)

Method	No. of Selected features	Accuracy	Precision	TPR_**c**_ (Recall)	FPR_**c**_	AUC
**Lasso + RF**	26	0.636	0.332	0.692	0.320	0.759
**Ridge + RF**	52	0.620	0.324	0.707	0.340	0.760
**ElasticNet + RF**	29	0.635	0.331	0.685	0.310	0.763
**LR + RF**	14	0.619	0.329	**0.718**	0.330	0.769
**MIE + RF**	42	0.642	0.334	0.684	0.310	0.759
**Caprini RAM**	**–**	**0.750**	**0.444**	0.667	**0.227**	**0.773**

### Model metrics of machine learning models based on EHR data

69 feature indicators are extracted from the EHR data to build the data-driven risk prediction models for VTE. For each feature, the MIE between itself and the target VTE is calculated to measure the strength of the feature’s importance. In the ranking of different features MIE values, the Caprini RAM score has the strongest correlation with VTE, besides other features extracted from EHR, like serum albumin (ALB), chemoprophylaxis, etc. (additional file 1).

Based on the preliminary observations, we use feature screening methods in machine learning such as Lasso regression, Ridge regression, ElasticNet regression, LR, and MIE separately to screen all the 69 features, in which Lasso, Ridge, ElasticNet, and LR are combined with the RFE framework, while MIE is combined with multicollinearity feature filtration. The number of features selected by the five screening methods ranges from 14 to 52, in which chemoprophylaxis, age, ISS, weight, pelvic injury, upper extremity injury, trauma history, red blood cell counts (RBC), and serum chloride level (CL) are commonly confirmed by all the methods (additional file 2).

Next, we carry out the machine learning modeling for predicting VTE according to the results of each feature screening method, which adopts the RF algorithm as the prediction model. The final model metrics are averaged on the testing sets, respectively. All the models testing metrics are shown in Table 2, and they are also compared to the testing metrics of the Caprini RAM.

**Fig. 3:**
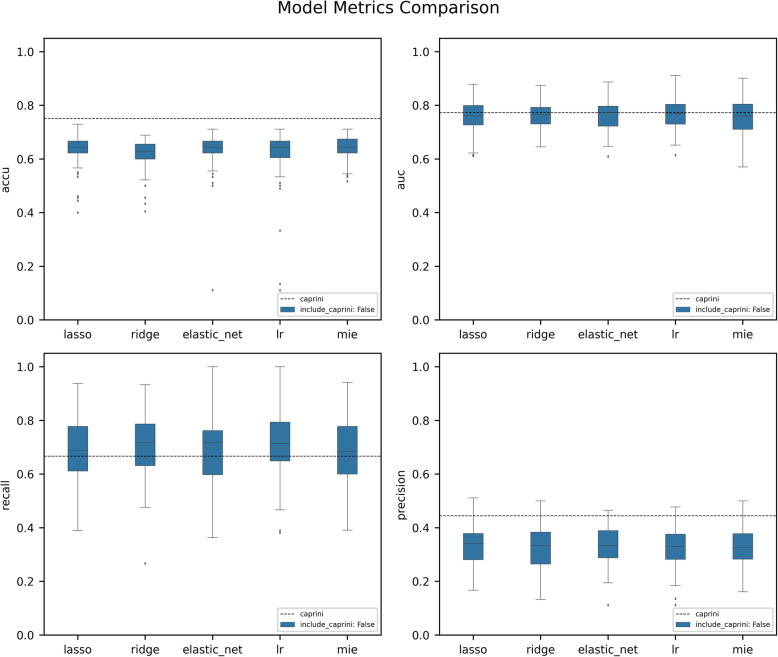
Prediction Metrics Distributions of Different Random Forest Models Based on EHR data

Figure 3 shows the distributions of the metrics from the above 5 RF models (blue box plots) compared with the Caprini RAM (horizontal dash line). It shows that the Caprini RAM gets better performance on metrics like accuracy, precision, FPR, and AUC, while the data-driven methods have slightly higher TPRs. In general, the Caprini RAM shows better prediction performance than RF models that are only based on EHR data.

### Model metrics of machine learning models based on Caprini scores and EHR data

We try to combine the Caprini RAM with the fields from the EHR data and perform the feature screening and modeling again to check if it is useful to get better model results. After adding the Caprini score, the feature screening results have some major changes. For Lasso and ElasticNet, the feature numbers decreased significantly to 5 and 6, while for Ridge, LR, and MIE, their modeling feature numbers change slightly. Features selected by all the five screening methods simultaneously are age, ISS, Caprini score, weight, and CL (additional file 2).

**Fig. 4:**
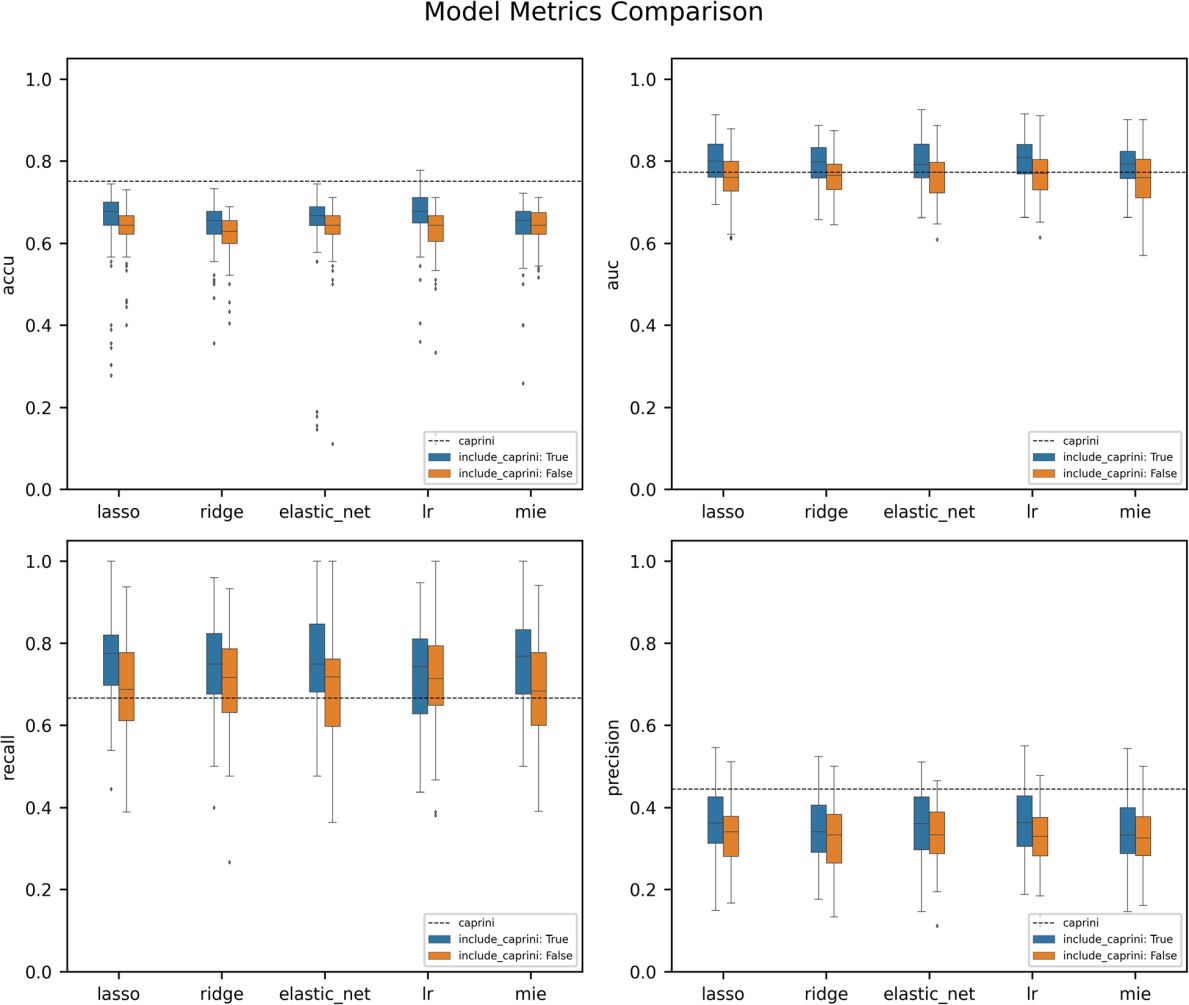
Prediction Metrics Distributions of Different Random Forest Models Based on EHR data & Caprini Scores

Similarly, we adopt a 10-fold validation framework to test all features set from the five screening methods and use the RF model to predict VTE. Metrics of each model are listed in Table 3. After adding the Caprini score into the features set, all the model prediction metrics get improved. Specifically, for group Caprini + MIE + RF, its TPR value reaches 0.780 and has increased a lot compared to the value 0.668 from Caprini RAM. However, gaps remain in Accuracy, Precision, and FPR between the data-driven random forest models and the Caprini RAM. Details are shown in Fig. 4.

**Table 3:** Prediction Metrics of Different Models based on EHR Data & Caprini Scores (Compared with the Caprini RAM)

Methods	No. of Selected features	Accuracy	Precision	TPR_**c**_ (Recall)	FPR_**c**_	AUC
**Caprini + Lasso + RF**	5	0.653	0.362	0.757	0.290	0.799
**Caprini + Ridge + RF**	50	0.643	0.346	0.746	0.320	0.792
**Caprini + ElasticNet + RF**	6	0.644	0.357	0.757	0.290	0.799
**Caprini + LR + RF**	13	0.671	0.366	0.722	0.270	**0.800**
**Caprini + MIE + RF**	43	0.637	0.344	**0.760**	0.330	0.793
**Caprini RAM**	**–**	**0.750**	**0.444**	0.667	**0.227**	0.773

**Fig. 5:**
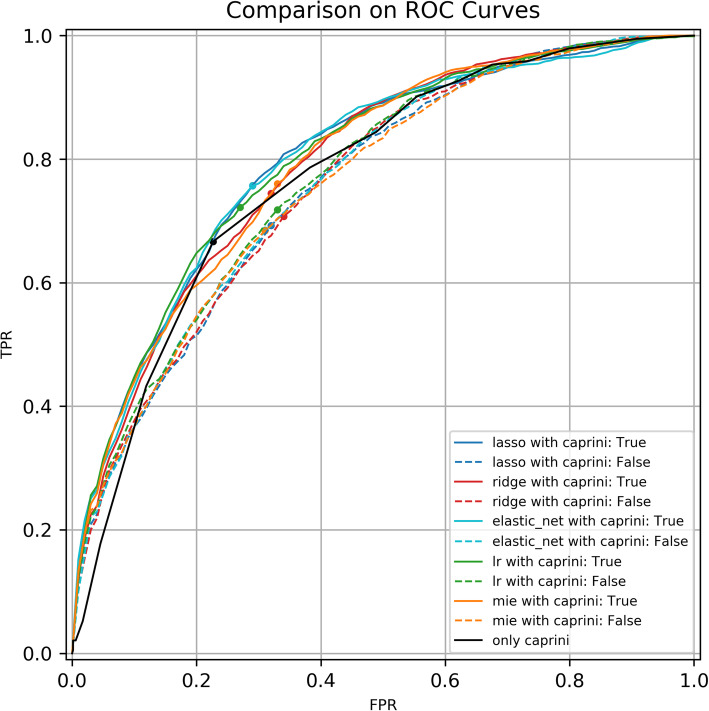
Comparisons on ROC curves and best cut-off points

Figure 5 shows the ROC curve and the corresponding best cut-off point for all the above models. It is obvious that the ROC of each model has been greatly improved after adding the Caprini scores.

In summary, comparing all the random forest models combining with the Caprini scores, there are no significant differences in metrics such as Accuracy, AUC, TPR, and FPR, but the group Caprini + Lasso + RF exploits the least features.

## Discussion

This paper verifies the effect of Caprini RAM in trauma patients for the first time and Caprini RAM shows good discrimination ability in predicting VTE events (TPR = 0.667, FPR = 0.227, AUC = 0.773). However, when divided by fraction, conventional risk stratification used in general surgical patients (0–1 point, 2 points, 3–4 points, ≥ 5 points) cannot effectively distinguish the risk of VTE in hospitalized trauma patients. Our research shows that VTE risk stratification based on 0–2 points, 3–6 points, 7–10 points, ≥ 11 points can be more appropriate for trauma patients. Existing research shows that there are differences in the cut-off point of Caprini RAM for different populations. The high-risk group of general surgical patients is defined by ≥5 points [14]. But for patients with hip fractures, some research shows that the best cut-off point is 12 points [20]. Dash J et al. [19] explored the cut-off point of Caprini RAM for lower extremity fracture is 12 overall. For total joint replacement, the cut-off point should be 10 points [31]. The ROC curve in this paper shows that the best cut-off point lies at 11 points, this tells us that for different disease populations, it is necessary to redefine the high-risk events and reassess the timing of chemical anticoagulation measures.

The effect of using anticoagulant measures for VTE prevention has already been recognized but as Defloor T. et al. [32] commented on pressure ulcer risk assessment, targeted preventive measures may reduce the risk of VTE in high-risk patients, reduce the actual VTE events, and reduce the sensitivity and specificity of prediction. In this study, a large number of patients used LMWH for anticoagulation therapy. It is reasonable to infer that the prediction accuracy of the risk assessment based on the Caprini score at the beginning of the patient’s admission should be better than our models’ accuracies.

In our research, physiological and biochemical indicators are directly incorporated into the VTE risk prediction models, because some research has shown that Caprini scores combined with serum markers can achieve better predictive performance [33]. Besides, using clinical and serological indicators is more helpful for computer-aided decision making [34]. Indicators that are unanimously selected in the feature screening processes with or without the Caprini scores include age, ISS, weight, and serum chloride (CL), in which the former 3 indicators are the same with the clinical experience and previous research [34–36], while the last CL is unexpectedly associated to the happening of VTE. Although there is no relevant literature report, considering that hyperchloremia is often associated with hypoproteinemia, hemoconcentration, renal insufficiency, and large amounts of fluid replacement, this association may be reasonable. It can be further verified in a prospective, large-sample study in the future.

When compared with machine learning models that only use features from EHR, Caprini RAM shows better prediction performance. But it is worthy to note that the Caprini RAM is a face-to-face evaluation of patients after admission and its score items are not completely included in the feature fields extracted by EHR, including recent sepsis, abnormal lung function, plaster fixation, family history of VTE, etc. It is reasonable to believe that the features extracted from the EHR cannot cover all the risk factors that Caprini RAM included. This may be one of the reasons why the prediction performance of the Caprini score is better than the performance of the machine learning models. Besides, the face-to-face evaluation of patients after admission may have higher data accuracy than the retrospective acquisition of feature fields from EHR [37].

There are certain differences in the prediction effects of the final constructed models, which are from different feature screening methods, but considering the risk of overfitting caused by the use of excessive features, we tend to choose the model with fewer features and better prediction performance. When only using EHR, the model LR + RF is more prominent and achieves the best TPR and the second-best AUC (worse than the AUC of Caprini RAM) with the least features. But when combining EHR and Caprini scores, all the models built by different feature screening methods achieve improved prediction performance with a reduction in the number of features. Although the Caprini + LR + RF model still keeps the highest AUC, its TPR and FPR are worse than those of other models. Here take the Caprini + Lasso + RF model, which only relies on 5 features, as an example. With the combination of EHR and Caprini scores, its sensitivity increases from 0.667 to 0.757 (the highest is 0.760), and AUC increased from 0.773 to 0.799 (the highest is 0.800), achieving a satisfying prediction effect. The Caprini score itself may be related to multiple features in EHR, and the L1 regularization used in Lasso regression can efficiently achieve dimensionality reduction of collinearity features, especially in high-dimensional sparse data [38]. It is shown in the screening results that the number of features decreased significantly after adding Caprini RAM into the data set. In summary, Caprini RAM can obtain better results when combined with Lasso + RF in feature screening and modeling.

Compared with all machine learning models, the Caprini RAM still keeps an advantage in Precision and FPR. When Caprini RAM is combined with machine learning models, it can identify the high-risk patients more effectively, while the Caprini model itself has a better ability in identifying low-risk patients. When it goes to guide VTE prevention, further considerations between risks caused by overtreatment and missed diagnosis should be confirmed by further diagnostic research.

As far as we know, this research is the first to focus on the effect of the Caprini RAM in trauma patients, which is rarely mentioned in previous studies. It can be concluded from the study that Caprini has good VTE prediction performance and discrimination in trauma patients, but its cut-off point is inconsistent with other diseases, and specific VTE risk stratification should be carried out for trauma patients.

We try to introduce the data extracted from EHR into the VTE prediction of trauma patients and verified that it has the potential to obtain better VTE prediction results when combined with the Caprini score and EHR data. Research shows that different feature screening methods will cause certain differences between the selected risk factors and the final modeling prediction effect. It is worth noting that as the number of features increases, the model is more likely to overfit, which leads to restrictions on external promotion. We suggest that, in feature selection, the number of features should be effectively reduced by regularization, to reduce the overuse of computing resources and the possibility of overfitting.

There are still some shortcomings in the research: (1) The study is retrospective, with small sample size and a single center. The model effect is cross-validated, and it has not to be evaluated in a multi-center, large-sample extensive trauma population; (2) Although we have focused on the occur of VTE in the patient’s hospital stay, we do not follow up with patients after discharge for a fixed period, which can lead to the missed screening of VTE events and underestimate the risk of VTE in trauma patients; (3) The data extracted from EHR cannot fully cover the Caprini score index, which may result in insufficient feature fields and affect the modeling effect of machine learning methods. Since the Caprini score is evaluated face-to-face when the patient is admitted to the hospital, the scores obtained can be more accurate than the data extracted from EHR. These two points may also be the reasons why the prediction effects of machine learning models are not as good as the Caprini RAM’s; (4) Although a variety of methods were used in feature screening, only RF is used in modeling, and other machine learning models such as support vector machines, gradient boosting machines, neural network, etc. are not evaluated, and no deep learning method was attempted. The above shortcomings will be further improved in future studies.

## Conclusion

Risk prediction plays an important role in clinical decision-making, and the introduction of machine learning methods provides exciting prospects for improving risk assessment and the formulation of targeted prediction strategies. Our results confirm the validity of the Caprini risk assessment scale and the improvement of the prediction effect of machine learning methods. In the future, we can further optimize the feature combination method of the existing VTE risk prediction scale through machine learning methods, or combine the clinical indicators available in the trauma database on this basis to improve the effect of VTE prediction. This will help the design of more automatic and efficient VTE risk assessment and decision support tools. We will try to further embed it in the electronic medical record system.

## Supplementary Information

**Additional file 1 Extract features characteristics**.

**Additional file 2 Feature selection results.**

## Data Availability

The datasets used or analyzed during the current study are not expected to be made available due to IRB approval restrictions but are available from the corresponding author on reasonable request.
