# Supplementary material for: Predicting venous thromboembolism in hospitalized trauma patients: a combination of the Caprini score and data-driven machine learning model
Source: BMC Emerg Med. 2021 May 10;21:60. doi: 10.1186/s12873-021-00447-x (PMC8111727; doi:10.1186/s12873-021-00447-x)
Supplement: Supplementary file 1 — Additional file 1 Extract features characteristics. [file 12873_2021_447_MOESM1_ESM.docx]

**APPENDIX 1. extract features characteristics**

**Table 1. extract features index**

| **Catergory** | **Label of Features** | **Features Description** | **Data Integrity** |
| --- | --- | --- | --- |
| Demographics | AGE | Age (years) | 100% |
|  | SEX | Gender (male/female) | 100% |
|  | HEIGHT | Height (cm) | 98.4% |
|  | WEIGHT | Weight (kg) | 95.0% |
|  | BMI | Body Mass Index (kg/m^2^) | 94.8% |
|  | ICU_STAY | ICU stay (yes/no) | 100% |
|  | SURGERY | Surgery (yes/no) | 100% |
|  | LOS | Length of hospital stay (days) | 100% |
|  | CAPRINI_SCORE | Caprini score (points) | 100% |
|  | CVC | central venous access (yes/no) | 100% |
|  | VTE* | DVT or PE (yes/no) | 100% |
|  | DAYS* | Days after Injury for VTE diagnosis (days) | 100% |
|  | CHEMOPROPHYLAXIS* | Chemoprophylaxis (yes/no) | 100% |
|  | LOS* | length of hospital stay (days) | 100% |
| Cormobidities and medical history | HBP | Hypertension (yes/no) | 100% |
|  | DM | Diabetes (yes/no) | 100% |
|  | COPD | Chronic obstructive pulmonary disease (yes/no) | 100% |
|  | HARMORRHAGIC_DISEASE | Bleeding disorder(yes/no) | 100% |
|  | PERIPHEARL_VASCULAR_DISEASE | peripheral vascular disease  (yes/no) | 100% |
|  | TUMOR | Cancer history (yes/no) | 100% |
|  | CORONARY_HEART_DISEASE | Coronary heart disease (yes/no) | 100% |
|  | HEART_FAILURE | Chronic heart failure (yes/no) | 100% |
|  | CKD | Chronic kidney disease (yes/no) | 100% |
|  | COGNITIVE_IMPAIRMENT | Cognitive impairment (yes/no) | 100% |
|  | PD | Parkinson’s disease (yes/no) | 100% |
|  | LIVER_CIRRHOSIS | Liver cirrhosis (yes/no) | 100% |
|  | STROKE | History of cerebrovascular disease  (yes/no) | 100% |
|  | TRAUMA_HISTORY | History of trauma (yes/no) | 100% |
|  | SMOKE | Current smoker (yes/no) | 100% |
|  | DRINKING | History of drinking (yes/no) | 100% |
|  | SURGERY_HISTORY | History of surgery (yes/no) | 100% |
|  | TRANSFUSION_HISTORY | History of transfusion (yes/no) | 100% |
| Laboratory examination results | RBC | Red blood cell count (10^12^/L) | 100% |
|  | HGB | Haemoglobin (g/L) | 100% |
|  | PLT | Platelet count (10^9^/L) | 100% |
|  | WBC | White blood cell count (10^9^/L) | 100% |
|  | ALB | Albumin (g/L) | 100% |
|  | CRE | Blood creatinine (μmol/L) | 100% |
|  | UA | Blood uric acid (μmol/L) | 100% |
|  | AST | Aspartate aminotransferase (U/L) | 100% |
|  | ALT | Alanine aminotransferase (U/L) | 100% |
|  | GLU | Blood glucose (mmol/L) | 100% |
|  | TG | Triglyceride (mmol/L) | 100% |
|  | CHO | Cholesterol (mmol/L) | 100% |
|  | LDL | Low density lipoprotein (mmol/L) | 100% |
|  | CA | Blood calcium (mmol/L) | 99.9% |
|  | MG | Blood magnesium (mmol/L) | 99.9% |
|  | NA | Blood sodium (mmol/L) | 99.9% |
|  | K | Blood potassium (mmol/L) | 99.9% |
|  | CL | Blood chlorine (mmol/L) | 99.8% |
|  | GFR | Glomerular filtration rate (ml/min) | 99.9% |
|  | PT | Prothrombin time (s) | 100% |
|  | INR | International Normalized Ratio | 100% |
|  | FIB | Fibrinogen (g/L) | 99.9% |
|  | DD | D-dimer (μg/L) | 95.9% |
|  | CK | Creatine kinase (U/L) | 100% |
| Vital signs | T | Temperature (℃) | 100% |
|  | P | Pulse rate (/min) | 100% |
|  | R | Breath (/min) | 100% |
|  | MBP | Mean arterial blood pressure (mmHg) | 100% |
|  | SHOCK_INDEX | Shock index | 100% |
| Injury Characteristics | INJURY_TYPE | injury mechanism  (blunt/penetrating) | 100% |
|  | INJURY_CAUSE | Injury cause (high fall/fall/traffic accident/crush/firearm/machine/sharp injury/others) | 100% |
|  | OPEN_INJURY | Open injury (yes/no) | 100% |
|  | HEAD | Head injury (yes/no) | 100% |
|  | THORACIC | Thoracic injury (yes/no) | 100% |
|  | ABDOMEN | Abdomen injury (yes/no) | 100% |
|  | SPINE | Spine injury (yes/no) | 100% |
|  | PELVIC | Pelvic injury (yes/no) | 100% |
|  | UPPER_EXTREMITY | Upper extremity injury (yes/no) | 100% |
|  | LOWER_EXTREMITY | Lower extremity injury (yes/no) | 100% |
|  | PARALYSIS | With Paralysis (yes/no) | 100% |
|  | ISS | Injury severity score (points) | 100% |

*: not included in VTE prediction model.


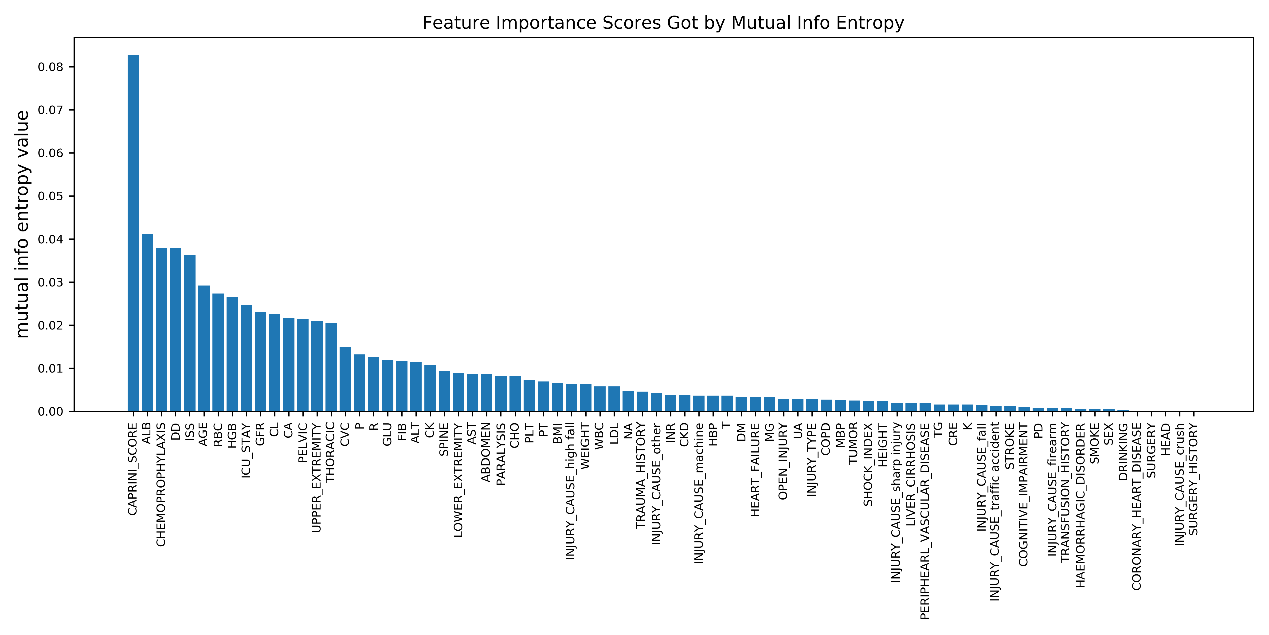


**Figure 1. Feature importance scores obtained by MIE**
