# Supplementary material for: Predicting venous thromboembolism in hospitalized trauma patients: a combination of the Caprini score and data-driven machine learning model
Source: BMC Emerg Med. 2021 May 10;21:60. doi: 10.1186/s12873-021-00447-x (PMC8111727; doi:10.1186/s12873-021-00447-x)
Supplement: Supplementary file 2 — Additional file 2 Feature selection results. [file 12873_2021_447_MOESM2_ESM.docx]

**APPENDIX 2. feature selection results**

**Table 1. Feature screening results from different methods (not including Caprini scores)***

| **feature** | **lasso** | **ridge** | **elastic_net** | **lr** | **mie** | **times been selected** |
| --- | --- | --- | --- | --- | --- | --- |
| **CHEMOPROPHYLAXIS** | 1 | 1 | 1 | 1 | 1 | 5 |
| **AGE** | 1 | 1 | 1 | 1 | 1 | 5 |
| **ISS** | 1 | 1 | 1 | 1 | 1 | 5 |
| **WEIGHT** | 1 | 1 | 1 | 1 | 1 | 5 |
| **PELVIC** | 1 | 1 | 1 | 1 | 1 | 5 |
| **UPPER_EXTREMITY** | 1 | 1 | 1 | 1 | 1 | 5 |
| **TRAUMA_HISTORY** | 1 | 1 | 1 | 1 | 1 | 5 |
| **RBC** | 1 | 1 | 1 | 1 | 1 | 5 |
| **CL** | 1 | 1 | 1 | 1 | 1 | 5 |
| **CVC** | 1 | 1 | 1 | 0 | 1 | 4 |
| **BMI** | 1 | 0 | 1 | 1 | 1 | 4 |
| **ICU_STAY** | 1 | 1 | 1 | 0 | 1 | 4 |
| **INJURY_CAUSE:high fall** | 1 | 1 | 1 | 0 | 1 | 4 |
| **LOWER_EXTREMITY** | 1 | 1 | 1 | 0 | 1 | 4 |
| **DM** | 1 | 1 | 1 | 0 | 1 | 4 |
| **WBC** | 1 | 1 | 1 | 0 | 1 | 4 |
| **ALB** | 1 | 0 | 1 | 1 | 1 | 4 |
| **GLU** | 1 | 1 | 1 | 0 | 1 | 4 |
| **CHO** | 1 | 1 | 1 | 0 | 1 | 4 |
| **PT** | 1 | 1 | 1 | 0 | 1 | 4 |
| **FIB** | 1 | 1 | 1 | 0 | 1 | 4 |
| **OPEN_INJURY** | 1 | 1 | 1 | 0 | 0 | 3 |
| **INJURY_CAUSE:fall** | 1 | 1 | 1 | 0 | 0 | 3 |
| **HEAD** | 1 | 1 | 1 | 0 | 0 | 3 |
| **SMOKE** | 1 | 1 | 1 | 0 | 0 | 3 |
| **TG** | 1 | 1 | 1 | 0 | 0 | 3 |
| **MG** | 0 | 1 | 0 | 1 | 1 | 3 |
| **NA** | 0 | 1 | 1 | 0 | 1 | 3 |
| T | 0 | 1 | 0 | 0 | 1 | 2 |
| P | 0 | 0 | 1 | 0 | 1 | 2 |
| INJURY_CAUSE**:**other | 0 | 1 | 0 | 0 | 1 | 2 |
| INJURY_CAUSE**:**machine | 0 | 1 | 0 | 0 | 1 | 2 |
| INJURY_CAUSE**:**firearm | 0 | 1 | 0 | 1 | 0 | 2 |
| THORACIC | 0 | 1 | 0 | 0 | 1 | 2 |
| ABDOMEN | 0 | 1 | 0 | 0 | 1 | 2 |
| SPINE | 0 | 1 | 0 | 0 | 1 | 2 |
| HEART_FAILURE | 0 | 1 | 0 | 0 | 1 | 2 |
| LIVER_CIRRHOSIS | 0 | 1 | 0 | 1 | 0 | 2 |
| CKD | 0 | 1 | 0 | 0 | 1 | 2 |
| PARALYSIS | 0 | 1 | 0 | 0 | 1 | 2 |
| DD | 0 | 0 | 1 | 0 | 1 | 2 |
| SEX | 0 | 1 | 0 | 0 | 0 | 1 |
| SURGERY | 0 | 1 | 0 | 0 | 0 | 1 |
| R | 0 | 0 | 0 | 0 | 1 | 1 |
| INJURY_TYPE | 0 | 1 | 0 | 0 | 0 | 1 |
| INJURY_CAUSE**:**crush | 0 | 1 | 0 | 0 | 0 | 1 |
| TRANSFUSION_HISTORY | 0 | 1 | 0 | 0 | 0 | 1 |
| SURGERY_HISTORY | 0 | 1 | 0 | 0 | 0 | 1 |
| PERIPHEARL_VASCULAR_DISEASE | 0 | 1 | 0 | 0 | 0 | 1 |
| TUMOR | 0 | 1 | 0 | 0 | 0 | 1 |
| HBP | 0 | 0 | 0 | 0 | 1 | 1 |
| COPD | 0 | 1 | 0 | 0 | 0 | 1 |
| HAEMORRHAGIC_DISORDER | 0 | 1 | 0 | 0 | 0 | 1 |
| CORONARY_HEART_DISEASE | 0 | 1 | 0 | 0 | 0 | 1 |
| COGNITIVE_IMPAIRMENT | 0 | 1 | 0 | 0 | 0 | 1 |
| PD | 0 | 1 | 0 | 0 | 0 | 1 |
| STROKE | 0 | 1 | 0 | 0 | 0 | 1 |
| PLT | 0 | 0 | 0 | 0 | 1 | 1 |
| AST | 0 | 0 | 0 | 0 | 1 | 1 |
| ALT | 0 | 0 | 0 | 0 | 1 | 1 |
| CA | 0 | 0 | 0 | 0 | 1 | 1 |
| LDL | 0 | 1 | 0 | 0 | 0 | 1 |
| GFR | 0 | 0 | 0 | 0 | 1 | 1 |
| CK | 0 | 0 | 0 | 0 | 1 | 1 |
| MBP | 0 | 0 | 0 | 0 | 0 | 0 |
| SHOCK_INDEX | 0 | 0 | 0 | 0 | 0 | 0 |
| HEIGHT | 0 | 0 | 0 | 0 | 0 | 0 |
| INJURY_CAUSE**:** traffic accident | 0 | 0 | 0 | 0 | 0 | 0 |
| INJURY_CAUSE**:** sharp injury | 0 | 0 | 0 | 0 | 0 | 0 |
| DRINKING | 0 | 0 | 0 | 0 | 0 | 0 |
| HGB | 0 | 0 | 0 | 0 | 0 | 0 |
| CRE | 0 | 0 | 0 | 0 | 0 | 0 |
| UA | 0 | 0 | 0 | 0 | 0 | 0 |
| K | 0 | 0 | 0 | 0 | 0 | 0 |
| INR | 0 | 0 | 0 | 0 | 0 | 0 |
| **TOTAL_NUM** | 26 | 52 | 29 | 14 | 42 | - |

*We use feature screening methods in machine learning such as Lasso regression, Ridge regression, Elastic Net regression , LR and MIE separately to screen all the 69 features, in which Lasso, Ridge, Elastic Net and LR are combined with the REF framework, while MIE is combined with multicollinearity (MC) feature filtration. The screening results are summarized in table 1, in which features chosen ≥ 3 times are taken as effective and denoted in bold and underlined. Number 1 means “chosen” and 0 means “dropped”.

**Table 2. Feature screening results from different methods (including Caprini scores )***

| **feature** | **lasso** | **ridge** | **elastic_net** | **lr** | **mie** | **times been selected** |
| --- | --- | --- | --- | --- | --- | --- |
| **AGE** | 1 | 1 | 1 | 1 | 1 | 5 |
| **ISS** | 1 | 1 | 1 | 1 | 1 | 5 |
| **CAPRINI_SCORE** | 1 | 1 | 1 | 1 | 1 | 5 |
| **WEIGHT** | 1 | 1 | 1 | 1 | 1 | 5 |
| **CL** | 1 | 1 | 1 | 1 | 1 | 5 |
| **CHEMOPROPHYLAXIS** | 0 | 1 | 0 | 1 | 1 | 3 |
| **BMI** | 0 | 1 | 0 | 1 | 1 | 3 |
| **TRAUMA_HISTORY** | 0 | 1 | 0 | 1 | 1 | 3 |
| **RBC** | 0 | 1 | 0 | 1 | 1 | 3 |
| **ALB** | 0 | 1 | 0 | 1 | 1 | 3 |
| **MG** | 0 | 1 | 0 | 1 | 1 | 3 |
| CVC | 0 | 1 | 0 | 0 | 1 | 2 |
| R | 0 | 1 | 0 | 0 | 1 | 2 |
| ICU_STAY | 0 | 1 | 0 | 0 | 1 | 2 |
| INJURY_CAUSE:high fall | 0 | 1 | 0 | 0 | 1 | 2 |
| INJURY_CAUSE:firearm | 0 | 1 | 0 | 1 | 0 | 2 |
| THORACIC | 0 | 1 | 0 | 0 | 1 | 2 |
| ABDOMEN | 0 | 1 | 0 | 0 | 1 | 2 |
| SPINE | 0 | 1 | 0 | 0 | 1 | 2 |
| PELVIC | 0 | 1 | 0 | 0 | 1 | 2 |
| UPPER_EXTREMITY | 0 | 1 | 0 | 0 | 1 | 2 |
| LOWER_EXTREMITY | 0 | 1 | 0 | 0 | 1 | 2 |
| HBP | 0 | 1 | 0 | 0 | 1 | 2 |
| DM | 0 | 1 | 0 | 0 | 1 | 2 |
| HEART_FAILURE | 0 | 1 | 0 | 0 | 1 | 2 |
| LIVER_CIRRHOSIS | 0 | 1 | 0 | 1 | 0 | 2 |
| CKD | 0 | 1 | 0 | 0 | 1 | 2 |
| WBC | 0 | 1 | 0 | 0 | 1 | 2 |
| GLU | 0 | 1 | 0 | 0 | 1 | 2 |
| CHO | 0 | 1 | 0 | 0 | 1 | 2 |
| PT | 0 | 1 | 0 | 0 | 1 | 2 |
| FIB | 0 | 1 | 0 | 0 | 1 | 2 |
| SEX | 0 | 1 | 0 | 0 | 0 | 1 |
| SURGERY | 0 | 1 | 0 | 0 | 0 | 1 |
| T | 0 | 0 | 0 | 0 | 1 | 1 |
| P | 0 | 0 | 0 | 0 | 1 | 1 |
| OPEN_INJURY | 0 | 1 | 0 | 0 | 0 | 1 |
| INJURY_TYPE | 0 | 1 | 0 | 0 | 0 | 1 |
| INJURY_CAUSE:fall | 0 | 1 | 0 | 0 | 0 | 1 |
| INJURY_CAUSE:traffic accident | 0 | 1 | 0 | 0 | 0 | 1 |
| INJURY_CAUSE:other | 0 | 0 | 0 | 0 | 1 | 1 |
| INJURY_CAUSE:crush | 0 | 1 | 0 | 0 | 0 | 1 |
| INJURY_CAUSE:machine | 0 | 0 | 0 | 0 | 1 | 1 |
| HEAD | 0 | 1 | 0 | 0 | 0 | 1 |
| SMOKE | 0 | 1 | 0 | 0 | 0 | 1 |
| DRINKING | 0 | 1 | 0 | 0 | 0 | 1 |
| PERIPHEARL_VASCULAR_DISEASE | 0 | 1 | 0 | 0 | 0 | 1 |
| TUMOR | 0 | 1 | 0 | 0 | 0 | 1 |
| COPD | 0 | 1 | 0 | 0 | 0 | 1 |
| HAEMORRHAGIC_DISORDER | 0 | 1 | 0 | 0 | 0 | 1 |
| CORONARY_HEART_DISEASE | 0 | 1 | 0 | 0 | 0 | 1 |
| PARALYSIS | 0 | 0 | 0 | 0 | 1 | 1 |
| HGB | 0 | 0 | 1 | 0 | 0 | 1 |
| PLT | 0 | 0 | 0 | 0 | 1 | 1 |
| AST | 0 | 0 | 0 | 0 | 1 | 1 |
| ALT | 0 | 0 | 0 | 0 | 1 | 1 |
| TG | 0 | 1 | 0 | 0 | 0 | 1 |
| CA | 0 | 0 | 0 | 0 | 1 | 1 |
| LDL | 0 | 1 | 0 | 0 | 0 | 1 |
| NA | 0 | 0 | 0 | 0 | 1 | 1 |
| K | 0 | 1 | 0 | 0 | 0 | 1 |
| GFR | 0 | 0 | 0 | 0 | 1 | 1 |
| DD | 0 | 0 | 0 | 0 | 1 | 1 |
| CK | 0 | 0 | 0 | 0 | 1 | 1 |
| MBP | 0 | 0 | 0 | 0 | 0 | 0 |
| SHOCK_INDEX | 0 | 0 | 0 | 0 | 0 | 0 |
| HEIGHT | 0 | 0 | 0 | 0 | 0 | 0 |
| INJURY_CAUSE:sharp injury | 0 | 0 | 0 | 0 | 0 | 0 |
| TRANSFUSION_HISTORY | 0 | 0 | 0 | 0 | 0 | 0 |
| SURGERY_HISTORY | 0 | 0 | 0 | 0 | 0 | 0 |
| COGNITIVE_IMPAIRMENT | 0 | 0 | 0 | 0 | 0 | 0 |
| PD | 0 | 0 | 0 | 0 | 0 | 0 |
| STROKE | 0 | 0 | 0 | 0 | 0 | 0 |
| CRE | 0 | 0 | 0 | 0 | 0 | 0 |
| UA | 0 | 0 | 0 | 0 | 0 | 0 |
| INR | 0 | 0 | 0 | 0 | 0 | 0 |
| **TOTAL_NUM** | 5 | 50 | 6 | 13 | 43 |  |

*After adding the Caprini score, the feature screening results have some major changes.
